# Predicting high lymph node positivity risk factors in nasopharyngeal carcinoma patients: A multi-model approach

**DOI:** 10.1097/MD.0000000000047162

**Published:** 2026-01-09

**Authors:** Hongming Liao, Benchao He, Fengbo Yan

**Affiliations:** a Department of Otolaryngology Head and Neck Surgery, Tianmen First People’s Hospital, Affiliated Hospital of Wuhan University of Science and Technology, Tianmen, Hubei Province, China.

**Keywords:** Surveillance, Epidemiology, and End Results Database, lymph node ratio, machine learning, nasopharyngeal carcinoma, regression Least Absolute Shrinkage and Selection Operator regression

## Abstract

Identifying patients at high risk of an elevated lymph node ratio (LNR) is critical for optimizing the management of nasopharyngeal carcinoma (NPC), as LNR, defined as the ratio of metastatic to examined lymph nodes, serves as a key prognostic indicator. This retrospective observational study aimed to investigate the epidemiology and influencing factors associated with high LNR in NPC patients. Various machine learning algorithms were employed to select independent predictive variables, and both univariate and multivariate Cox regression analyses were conducted to develop predictive models. The performance of different models was evaluated using receiver operating characteristic curves, calibration plots, and decision curve analysis, and nomograms and survival curves were constructed to facilitate visualization and clinical interpretation. A total of 1563 NPC patients were included in the study. The optimal model demonstrated an area under the curve of 0.73 (95% confidence interval: 0.67–0.78) in the modeling group and 0.76 (95% confidence interval: 0.70–0.81) in the validation group. The nomogram identified N stage, M stage, type of surgery, race, and confirmation status as independent risk factors for high LNR. Survival curve analysis further indicated that patients classified as high-risk by the nomogram had significantly worse outcomes. These findings suggest that elevated LNR is strongly associated with adverse prognosis in NPC patients. The constructed nomogram serves as a practical clinical tool to stratify patients based on LNR risk, thereby enabling personalized follow-up, treatment planning, and management strategies to optimize patient outcomes.

## 1. Introduction

Nasopharyngeal carcinoma (NPC) is a prevalent malignant tumor primarily affecting individuals in southern China. Its pathogenesis is multifactorial, involving Epstein-Barr virus (EBV) infection, carcinogen exposure, and individual susceptibility. Clinically, NPC is characterized by poor differentiation and higher metastasis rates compared to other head and neck cancers. Despite advancements in chemotherapy and radiotherapy, treatment outcomes for NPC remain suboptimal, with persistent challenges such as local recurrence and distant metastases.^[1,2]^

At diagnosis, approximately 70% to 80% of NPC patients present with cervical lymph node metastases.^[3,4]^ Lymph node metastasis significantly impacts NPC treatment and prognosis, notably affecting 5-year survival rates.^[5]^ Specifically, around 60% of stage III-IV NPC cases exhibit regional lymph node involvement at diagnosis.^[6]^ Although combined therapeutic approaches have improved the 5-year survival rate from approximately 50% to 70%,^[7,8]^ outcomes remain variable.

Currently, tumor-node-metastasis staging serves as the primary prognostic tool for NPC. However, this anatomically based system may lack sensitivity in distinguishing between patients with NPC.^[9]^ Recent updates to the American Joint Committee on Cancer and Union for International Cancer Control staging systems underscore the prognostic importance of the N stage.^[10]^ Additionally, alternative scoring systems, such as the lymph node ratio (LNR), the ratio of positive lymph nodes to the total lymph nodes resected, have been investigated to enhance prognostic accuracy.^[11,12]^ The LNR metric has demonstrated superior prognostic value compared to traditional number-based staging systems.

This study aims to utilize clinical data from the Surveillance, Epidemiology, and End Results (SEER) database to analyze and develop predictive models for identifying high-risk NPC patients based on LNR. Our goal is to evaluate how prognostic indicators influence survival outcomes in NPC patients.

## 2. Materials and methods

### 2.1. Recruitment of patients from the SEER database

The SEER program, established by the National Cancer Institute,^[13,14]^ has been crucial in exploring clinical variables related to NPC from 2004 to 2015. This program provides essential clinical data for patients diagnosed with NPC during this timeframe.

**Figure 1.**
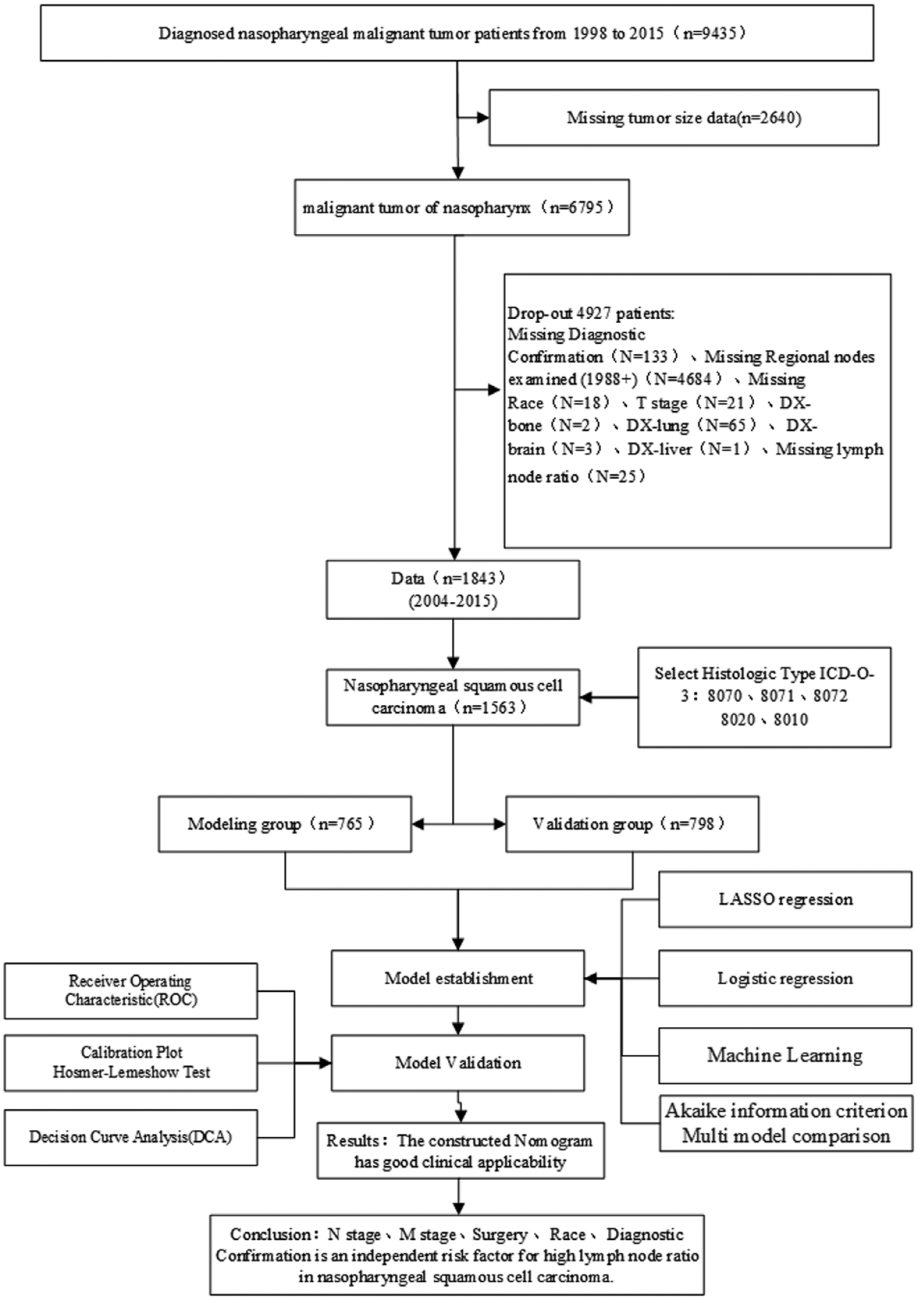
The inclusion criteria flowchart of recruited patients in SEER database. SEER = Surveillance, Epidemiology, and End Results Database.

The study’s inclusion criteria were as follows: a confirmed diagnosis of nasopharyngeal carcinoma (ICD-O-3 site recode); availability of complete and accurate basic clinical information; and assessment of both the number of positive lymph nodes and the total number of lymph nodes. Cases meeting these criteria were randomly allocated into modeling and validation groups, ensuring equal sample sizes for each group. A detailed description of the screening process is illustrated in the flowchart provided in Figure 1. This study was approved by the Ethics Committee of Tianmen First People’s Hospital (Approval No. 20240246).

### 2.2. Clinical variables extracted for analysis

The study gathered a range of demographic and clinical variables, including sex, age at diagnosis, race, marital status, tumor grade, diagnostic confirmation, tumor stage (T stage), nodal stage (N stage), metastatic status (M stage), surgical treatment, presence of distant metastases (e.g, bone, brain, liver, and lung), primary indicator, sequence number, survival duration in months, vital status, year of diagnosis, and the number of regional lymph nodes examined and identified as positive.

The LNR was calculated by dividing the number of positive lymph nodes by the total number of lymph nodes examined.^[15]^ The threshold value distinguishing high from low LNR categories was determined using the receiver operating characteristic (ROC) curve.

The primary outcome measure for this study was overall survival (OS), defined as the interval between the date of diagnosis and the date of death from any cause.

### 2.3. Establish different models for comparison

Initially, we conducted univariate and multivariate logistic regression analyses using the backward elimination method on all independent variables. From these analyses, we identified 5 variables that significantly impacted the outcome, which were then used to develop Model A through multiple logistic regression.

Subsequently, we employed 5 machine learning algorithms, decision tree, random forest, gradient boosting machine, Lasso regression, and XGBoost, to identify 7 key independent variables by intersecting their results. These 7 variables were then utilized to construct Model B through multiple logistic regression.

For Model C, Lasso regression was applied to all independent variables, selecting those associated with the minimum error. In Model D, variables were chosen based on a 1 standard error (1SE) criterion to construct the model. The optimal model was determined by comparing the Akaike Information Criterion (AIC) across different models, with the selected model used to develop the Nomogram.

### 2.4. Construction and validation of the nomogram

To evaluate the accuracy and discriminatory performance of the nomogram, we utilized both the ROC curve and the calibration curve. The area under the ROC curve (AUC) measures the model’s ability to discriminate between outcomes, while the Hosmer-Lemeshow test assesses its calibration accuracy. Additionally, decision curve analysis (DCA) and clinical impact curve (CIC) were employed to determine the model’s clinical utility by evaluating the net benefit at various risk thresholds. DCA specifically helped in assessing the practical applicability of the nomogram in a clinical setting.

### 2.5. Statistical analysis

Statistical analyses were conducted using SPSS version 25.0 (SPSS, Chicago, IL, USA), R software (version 4.1.2), and GraphPad Prism version 8.0 (GraphPad, Inc.). The “glmnet” package in R was employed to implement the LASSO algorithm. Normality of continuous variables was tested, with nonparametric tests applied where normality assumptions were not met. Continuous variables are reported as mean ± standard deviation (SD), while categorical variables are expressed as frequency (percentage). Group differences were evaluated using the chi-square test or Fisher exact test, with a significance level set at *P* < .05. Kaplan–Meier survival curves and log-rank tests were used to compare survival between groups.

## 3. Results

### 3.1. Baseline characteristics of the patient

A total of 1563 eligible NPC patients from the SEER database were included in the study. Of these, 765 patients were randomly allocated to the Modeling group, while 798 patients were assigned to the Validation group. No significant differences were found between the Modeling and Validation groups across any of the included variables (Table 1).

**Table 1:** Comparison of general information between modeling and validation groups.

Characteristics	Category	Modeling group		Validation group		*P*
		(N = 765)	ratio(%)	(N = 798)	Ratio(%)	
Sex	Female	212	27.71	226	28.32	.789
	male	553	72.29	572	71.68	
Race	White	357	46.67	393	49.25	.263
	Black	87	11.37	72	9.02	
	Other	321	41.96	333	41.73	
Marital status	Married	429	56.08	478	59.9	.126
	Unmarried	336	43.92	320	40.1	
Grade	I–II	61	7.97	51	6.39	.462
	III–IV	398	52.03	417	52.26	
	Unknown	306	40	330	41.35	
Confirmation	Positive histology	682	89.15	722	90.48	.386
	Other	83	10.85	76	9.52	
T stage	T0–T2	447	58.43	496	62.16	.132
	T3、T4、Tx	318	41.57	302	37.84	
N stage	N0-N1	334	43.66	348	43.61	.984
	N2-N3	431	56.34	450	56.39	
M stage	M0	609	79.61	641	80.33	.723
	M1	156	20.39	157	19.67	
Surgery	No	680	88.89	702	87.97	.57
	Yes	85	11.11	96	12.03	
Bone	No	349	45.62	366	45.86	.955
	Yes	31	4.05	30	3.76	
	Blank(s)	385	50.33	402	50.38	
Brain	No	377	49.28	393	49.25	1*
	Yes	3	0.39	3	0.38	
	Blank(s)	385	50.33	402	50.38	
Liver	No	361	47.19	380	47.62	.813
	Yes	19	2.48	16	2.01	
	Blank(s)	385	50.33	402	50.38	
Lung	No	364	47.58	380	47.62	.993
	Yes	16	2.09	16	2.01	
	Blank(s)	385	50.33	402	50.38	
Primary indicitor	No	65	8.5	55	6.89	.234
	Yes	700	91.5	743	93.11	
Number	1	644	84.18	697	87.34	.074
	≥2	121	15.82	101	12.66	
Age		53(44–64)		52(42–62)		.1

* Fisher exact test.

### 3.2. Comparison of NPC mortality rates and the high proportion of LNR in different years

**Figure 2.**
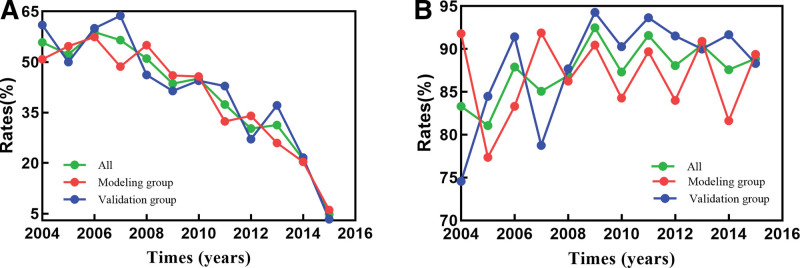
(A) Comparison of NPC mortality rates in different years. (B) Comparison of high LNR proportion in different years. LNR = lymph node ratio, NPC = nasopharyngeal carcinoma.

We calculated the annual mortality rate for patients with NPC as a percentage of the total population, which showed a downward trend over time. The peak mortality rate was observed in 2006 at 58.87%, while the lowest rate was recorded in 2015 at 4.76%. Additionally, we assessed the annual percentage of patients with high LNR within the total population. The proportion of high-LNR patients remained relatively stable across years, with the highest proportion of 92.48% recorded in 2009 and the lowest of 81.08% in 2005 (Fig. 2).

### 3.3. Identification of independent prognostic factors for high lymph node ratio in the modeling group

**Figure 3.**
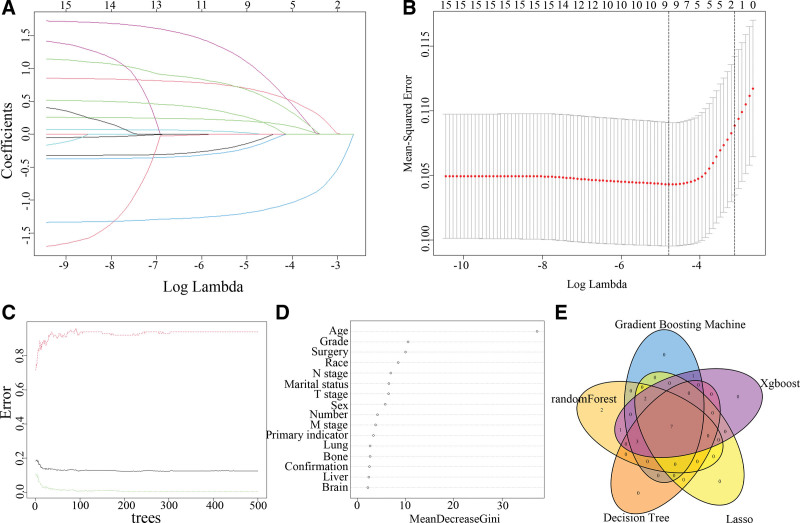
Selection of independent variables using different methods. (A and B) LASSO regression screening for independent variables. (C) The relationship between trees and errors in Random Forests. (D) Variable importance plot of the RFl for predicting NPC infection risk. (E) Obtain intersection independent variables through 5 machine learning algorithms (decision tree, random forest, gradient boosting machine, Lasso regression, and XGBoost). LASSO = regression Least Absolute Shrinkage and Selection Operator regression, NPC = nasopharyngeal carcinoma.

In the univariate analysis, significant associations were observed between confirmation, T stage, N stage, M stage, surgical intervention, lymph node count, race, and tumor grade. Subsequently, multivariate analysis using backward logistic regression identified race, surgery, N stage, M stage, and confirmation as independent risk factors (Table 2). These variables were included in Model A. Meanwhile, Lasso regression, based on the minimum lambda, selected 9 independent variables: sex, race, marital status, tumor grade, confirmation, N stage, M stage, surgery, and primary indicator. Models C and D were constructed using multivariate logistic regression with 2 independent variables (N stage and surgery) at 1 standard error (1se) (Fig. 3A and 3B). The random forest algorithm was then applied to assess the relationship between error and the variables (Fig. 3C), as well as to evaluate the importance of each variable in predicting outcomes (Fig. 3D). Using 5 machine learning algorithms, decision tree, random forest, gradient boosting machine, Lasso regression, and XGBoost, we identified 7 independent variables: N stage, M stage, sex, race, marital status, confirmation, and surgery. These variables were used to construct Model B, a multivariate logistic regression model (Fig. 3E).

**Table 2:** Univariate and multivariate analysis of factors related to high LNR in nasopharyngeal carcinoma patients.

Characteristics	Univariate analysis			multivariate analysis		
	OR	95%CI	*P*	OR	95%CI	*P*
Sex	0.83	0.51–1.35	.45			
Age	1	0.99–1.01	.98			
Marital status	0.79	0.52–1.21	.28			
Confirmation	6.64	1.61–27.43	.01	5.37	1.28–22.49	.02
T stage	1.33	0.85–2.06	.21			
N stage	2.5	1.61–3.88	0	2.23	1.41–3.52	0
M stage	2.8	1.38–5.68	.01	2.45	1.18–5.1	.02
Surgery	0.23	0.14–0.39	0	0.28	0.16–0.48	0
Primary indicitor	1.62	0.83–3.15	.16			
Number	0.6	0.36–1.01	.06			
Race			0	0	0	.07
Race (White)	0.47	0.3–0.76	0	0.58	0.35–0.94	.03
Race (Black)	1.14	0.48–2.69	.77	0.96	0.39–2.35	.93
Grade			.02			
Grade (I~II)	0.38	0.19–0.76	.01			
Grade (III~IV)	0.89	0.56–1.42	.63			
bone			.55			
Bone (No)	0.94	0.61–1.44	.77			
Bone (Yes)	2.12	0.49–9.14	.32			
brain			.99			
Brain (No)	0.98	0.64–1.49	.91			
liver			.62			
Liver (No)	0.95	0.62–1.46	.82			
Liver (Yes)	2.63	0.34–20.1	.35			
lung			1			
Lung (No)	0.98	0.64–1.51	.94			
Lung (Yes)	1.02	0.23–4.63	.98			

### 3.4. Evaluation and selection of the optimal model

The cutoff value for the LNR was determined to be 0.525 based on ROC curve analysis using overall survival as the endpoint. The optimal threshold corresponding to the maximum Youden index was selected, yielding a sensitivity of 0.316, a specificity of 0.778, and an AUC of 0.541 (95% confidence interval [CI]: 0.468–0.614). Given that the LNR ranges from 0 to 1, this cutoff allowed us to categorize patients into 2 distinct groups: high LNR and low LNR, which were used as outcome variables in this study.

To determine the best-performing model, we compared the AIC values for Models A, B, C, and D. The comparison between Model A and Model B yielded a χ² value of 3.96 with a *P*-value of .1383, indicating no significant difference in performance (*P* > .05). Consequently, Model A was deemed optimal. Similarly, when comparing Model A with Model C (χ² = 5.56, *P* = .2345), the *P*-value exceeded .05, suggesting that Model A and Model C performed similarly, reaffirming Model A as the optimal choice. In contrast, the comparison of Model A with Model D (χ² = 21.75, *P* = .0001) showed a *P*-value <.05, indicating a significant difference in performance. Given that the optimal model is defined by a lower AIC, Model A was ultimately selected as the best model (Table 3).

**Table 3:** Comparison between different models.

Model	N	Df	AIC	BIC
ModelA	765	6	532.5763	560.4155
ModelB	765	8	532.6193	569.7383
ModelC	765	10	535.0186	581.4173
ModelD	765	3	548.3299	562.2495

### 3.5. Creation and validation of nomograms

#### 3.5.1. Model discrimination

**Figure 4.**
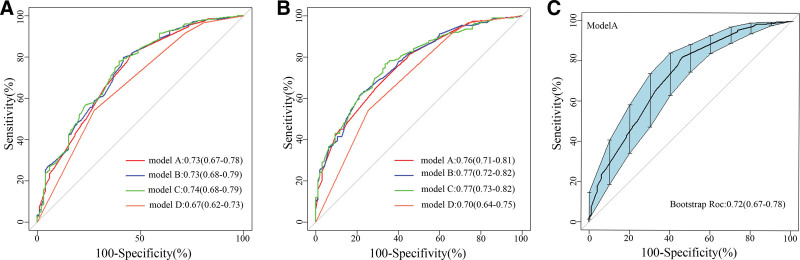
ROC curves of different models. (A) Modeling group; (B) validation group; (C) ROC curve with 1000-times bootstrap cross-validation. ROC = receiver operating characteristics curve.

ROC curves were constructed for Models A, B, C, and D using multivariate logistic regression, both for the modeling and validation groups (Fig. 4). In the modeling group, the AUCs were as follows: Model A, 0.73 (95% CI: 0.67–0.78); Model B, 0.73 (95% CI: 0.68–0.79); Model C, 0.74 (95% CI: 0.68–0.79); and Model D, 0.67 (95% CI: 0.62–0.73). In the validation group, the AUCs were: Model A, 0.76 (95% CI: 0.71–0.81); Model B, 0.77 (95% CI: 0.72–0.82); Model C, 0.77 (95% CI: 0.72–0.82); and Model D, 0.70 (95% CI: 0.64–0.75).To reduce the risk of overfitting during variable selection and model development, a 1000-time bootstrap cross-validation was conducted for the final Model A. The results confirmed that the model maintained satisfactory robustness, with an AUC of 0.72 (95% CI: 0.67–0.78), as presented in Figure 4C. These findings demonstrate that all models performed well in distinguishing between high and low LNR categories, as evidenced by the ROC curve analyses. Detailed indicators for each model’s ROC curve are summarized in Table 4.

**Table 4:** Relevant indicators of ROC curves for each model.

model	Cutoff	AUC	ACC	SEN	SPE	PLR	NLR	PPV	NPV	KAPPA
modeling A	0.791	0.725 (0.669–0.781)	0.783 (0.783–0.783)	0.819 (0.789–0.848)	0.541 (0.442–0.639)	1.783 (1.434–2.217)	0.335 (0.263–0.428)	0.924 (0.902–0.945)	0.305 (0.236–0.373)	0.27 (0.19–0.351)
modeling B	0.819	0.733 (0.678–0.789)	0.769 (0.768–0.769)	0.796 (0.766–0.827)	0.582 (0.484–0.679)	1.903 (1.502–2.411)	0.351 (0.28–0.439)	0.928 (0.907–0.949)	0.295 (0.231–0.36)	0.267 (0.19–0.344)
modeling C	0.839	0.738 (0.682–0.793)	0.757 (0.756–0.757)	0.78 (0.748–0.811)	0.602 (0.505–0.699)	1.959 (1.531–2.507)	0.366 (0.295–0.454)	0.93 (0.909–0.951)	0.286 (0.225–0.348)	0.26 (0.185–0.334)
modeling D	0.891	0.671 (0.615–0.727)	0.565 (0.564–0.565)	0.541 (0.503–0.579)	0.724 (0.636–0.813)	1.964 (1.414–2.729)	0.633 (0.546–0.734)	0.93 (0.905–0.956)	0.188 (0.149–0.228)	0.12 (0.072–0.168)
validation A	0.873	0.759 (0.71–0.809)	0.653 (0.652–0.653)	0.644 (0.609–0.68)	0.716 (0.625–0.806)	2.267 (1.64–3.134)	0.497 (0.423–0.584)	0.944 (0.923–0.964)	0.214 (0.169–0.259)	0.179 (0.123–0.234)
validation B	0.89	0.768 (0.719–0.816)	0.632 (0.631–0.632)	0.61 (0.574–0.646)	0.789 (0.707–0.871)	2.899 (1.955–4.298)	0.494 (0.43–0.567)	0.955 (0.936–0.975)	0.215 (0.172–0.258)	0.185 (0.134–0.237)
validation C	0.849	0.773 (0.726–0.821)	0.753 (0.753–0.754)	0.765 (0.734–0.797)	0.663 (0.568–0.758)	2.272 (1.708–3.021)	0.354 (0.291–0.43)	0.944 (0.925–0.963)	0.276 (0.218–0.334)	0.267 (0.196–0.337)
validation D	0.891	0.696 (0.639–0.753)	0.565 (0.565–0.566)	0.541 (0.504–0.577)	0.747 (0.66–0.835)	2.14 (1.504–3.044)	0.615 (0.534–0.708)	0.941 (0.918–0.964)	0.18 (0.142–0.218)	0.122 (0.077–0.167)

#### 3.5.2. Model calibration

**Figure 5.**
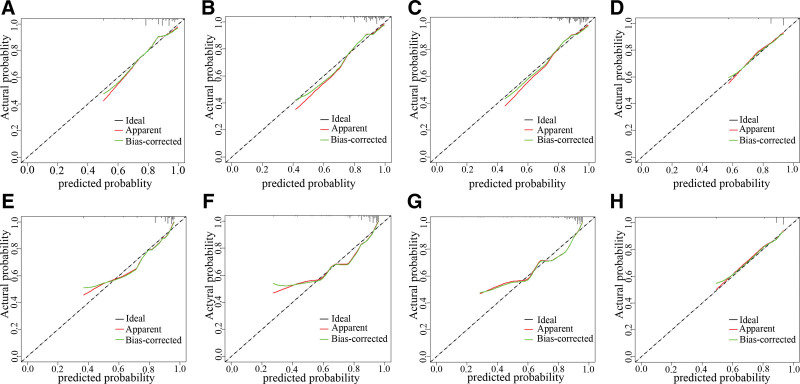
Calibration curves for different models. (A–D) Modeling group; (E–H) validation group.

Calibration curves for Models A, B, C, and D were constructed for both the modeling and validation groups, and Hosmer-Lemeshow tests were performed to evaluate model calibration. The calibration curves demonstrated robust calibration for both groups. In the modeling group, the calibration results were as follows: Model A (χ² = 4.77, *P* = .85), Model B (χ² = 8.92, *P* = .44), Model C (χ² = 9.78, *P* = .37), and Model D (χ² = 0.07, *P* = 1.00). For the validation group, the calibration results were: Model A (χ² = 4.72, *P* = .86), Model B (χ² = 6.85, *P* = .65), Model C (χ² = 6.15, *P* = .72), and Model D (χ² = 1.66, *P* = 1.00). These results indicate that all models demonstrate satisfactory calibration performance, as shown in Figure 5.

#### 3.5.3. Clinical applicability of the model and nomogram

**Figure 6.**
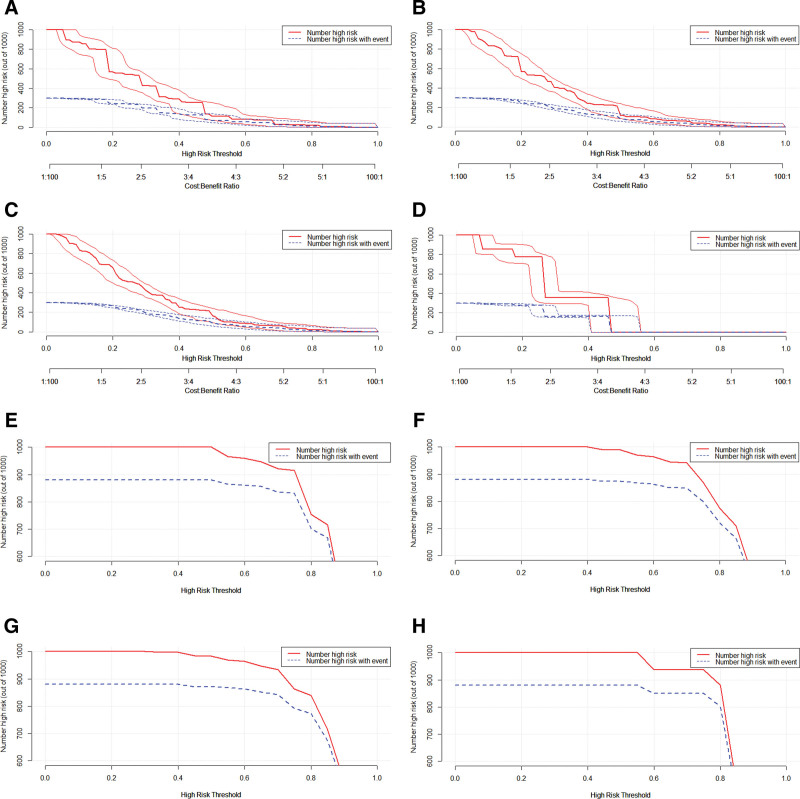
Clinical impact curve for different models. (A–D) Modeling group; (E–H) validation group.

Clinical impact curves were generated for both the modeling and validation groups to assess the practical utility of the models. In these curves, the solid red line represents the high-risk population as predicted by the model, while the dashed blue line indicates the actual number of individuals experiencing risk events across different thresholds. A closer alignment of these lines signifies greater model effectiveness. The clinical impact curve for the modeling group demonstrated superior efficiency compared to the validation group, as illustrated in Figure 6.

**Figure 7.**
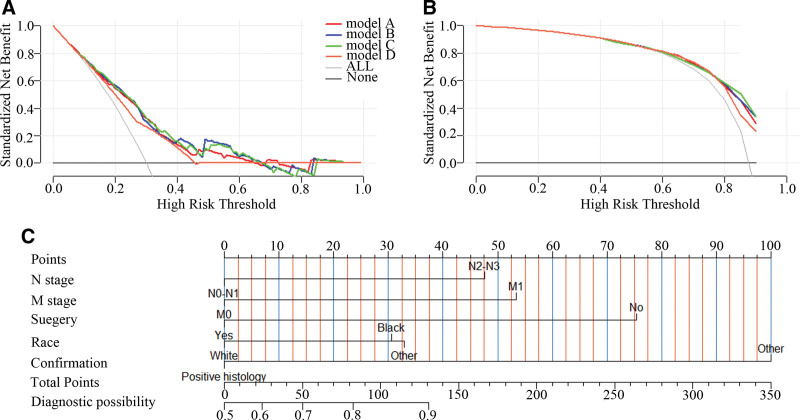
Decision curve analysis for different models and Nomogram of Model A. (A) modeling group; (B) validation group; (C) Nomogram.

Additionally, decision curve analysis was conducted, revealing that both the modeling and validation groups display good clinical applicability within certain thresholds, as shown in Figure 7A and 7B.

Finally, a nomogram based on Model A was developed, identifying N stage, M stage, surgery, race, and confirmation as independent risk factors for high LNR in NPC patients, as depicted in Figure 7C.

### 3.6. Survival curves

**Figure 8.**
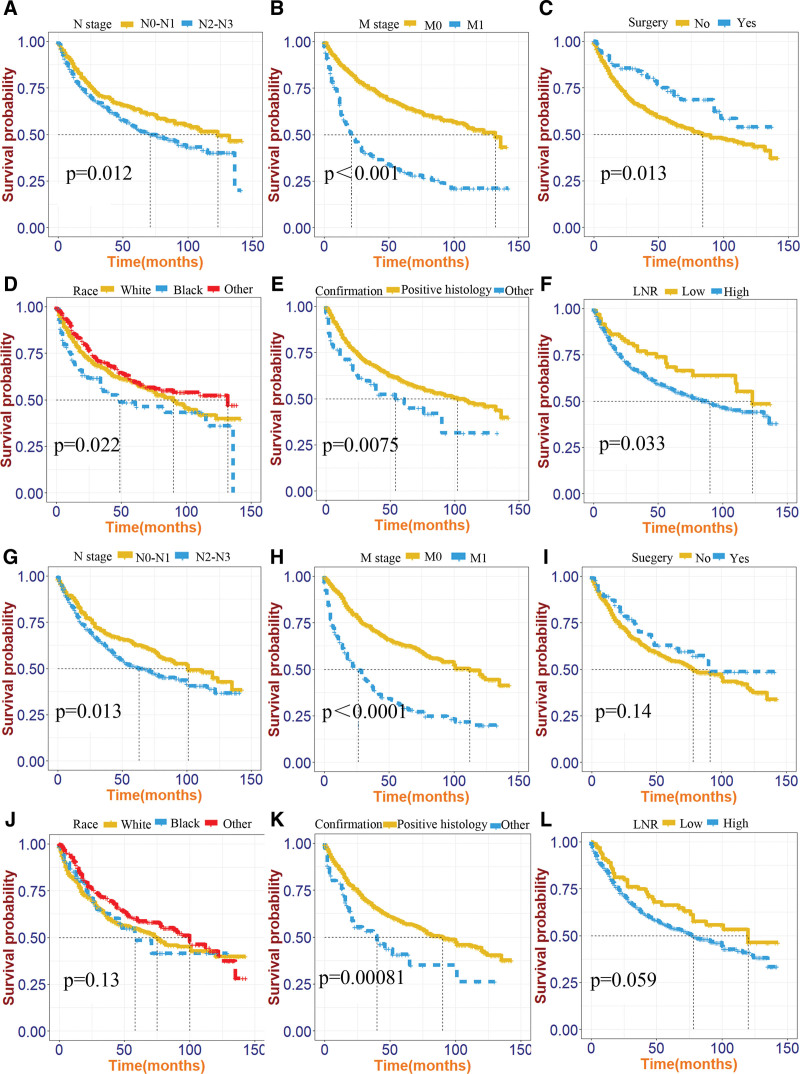
Survival curves for different variables. (A–F) modeling group; (G–I) validation group.

Survival curves were plotted for each variable in both the modeling and validation groups, based on survival time and status. In the modeling group, statistically significant differences were observed in the survival curves for stage N, stage M, surgery, race, confirmation, and LNR (*P* < .05). Similarly, in the validation group, significant differences were noted for stage N, stage M, and confirmation (*P* < .05). These results are illustrated in Figure 8.

## 4. Discussion

NPC is a highly prevalent malignancy in Southeast Asia, particularly in South China.^[16–18]^Although its etiology is complex, the precise cause of NPC remains unclear. Research has highlighted several factors contributing to NPC development, including dietary influences, infections with carcinogenic viruses such as EBV, and genetic predisposition.^[19]^With the rapid advancements in radiotherapy and chemotherapy, the long-term survival rates for NPC patients have markedly improved.^[20–22]^However, NPC is often asymptomatic in its early stages and frequently presents with cervical lymph node metastasis.^[23–25]^This highlights the critical need for accurate evaluation of lymph node metastasis to guide optimal treatment strategies.

In our study, the LNR was identified as a significant independent prognostic indicator for cancer-specific survival.^[26,27]^While categorizing a continuous variable like LNR may lead to potential data loss and measurement inaccuracies, its simplicity and ease of interpretation make it advantageous for routine clinical application.^[28]^It is essential to establish appropriate cutoff points in LNR classification to maintain consistency across studies and ensure adequate representation of individuals and events within each group.^[29]^ We determined the cutoff value for LNR using the ROC curve and classified LNR into high and low groups accordingly. This classification served as the outcome variable to examine how each independent factor influenced the likelihood of a high LNR. By constructing and comparing various models in terms of discrimination, calibration, clinical applicability, and Akaike information criteria, we identified N stage, M stage, surgery, race, and confirmation as independent risk factors for high LNR.Consistent with our findings, another study analyzing cervical recurrent nasopharyngeal carcinoma patients treated at Mary Hospital between 2000 and 2011 reported that LNR was the only independent predictor of nodal recurrence (*P* = .045) and nodal recurrence-free survival (*P* = .010). Although LNR was significantly associated with overall survival in univariate analysis (*P* = .001), its significance diminished in multivariate analysis when local recurrence was included. Moreover, that study identified 10% and 15% as effective cutoff values for stratifying patients into low-, intermediate-, and high-risk groups (*P* = .02), further supporting the prognostic value of LNR in NPC.^[30]^Recent evidence indicates that nomograms incorporating regional lymph node density (RLND) can effectively predict prognosis and stratify risk in NPC patients without distant metastases.^[31]^ In a cohort of 610 patients (425 in the training cohort and 185 in the validation cohort), MRI-assessed nodal features and clinical data were collected, and RLND was calculated. Multivariate Cox analysis identified independent prognostic factors for constructing the nomograms. Evaluation using C-index, ROC, calibration curves, and decision curve analysis confirmed RLND as an independent predictor of overall survival and disease-free survival (hazard ratios 1.36 and 1.30). Compared with previous predictive models, such as RLND-based assessments alone, the nomograms developed in the current study integrate additional clinical and pathological factors, thereby enhancing discrimination and calibration and providing a more individualized risk stratification approach for NPC patients.

This study benefits from the extensive sample size provided by the SEER database. The SEER data, retrospectively extracted from a registry that covers 26% of the U.S. population, is considered representative and helps to minimize selection bias, recall bias, treatment trends, and the impact of loss to follow-up on study outcomes. Additionally, it reduces the risk of overlooking data that might arise when collecting information from individual institutions.^[32]^However, despite these advantages, variations in patient management practices across institutions and the lack of certain details in pathological reports and covariates may introduce potential biases when analyzing SEER data.Importantly, the nomograms constructed in this study not only predict survival risks associated with LNR but also provide clear clinical utility. Specifically, clinicians can use the nomogram to adjust radiotherapy dose or target volumes based on the assessed high- or low-risk status; for high-risk patients, additional adjuvant treatments such as chemotherapy, targeted therapy, or immunotherapy may be considered; meanwhile, the nomogram can guide individualized follow-up schedules, including the frequency of examinations and imaging assessments. These practical applications link the predictive results directly to clinical management, offering more refined and personalized strategies for NPC patients.

Biomedical researchers are increasingly adopting machine learning (ML) techniques to enhance their analyses. ML-based algorithms have shown great promise in applications such as screening, diagnostics, and prognostics.^[33]^In this study, we utilized machine learning algorithms to filter independent variables. Tree-based algorithms, like decision trees, are particularly valued for their simplicity and high accuracy in predictive tasks. As part of the supervised learning category, these algorithms learn from the data by classifying subjects based on assigned categories.^[34]^Given that our input data includes both categorical and nominal features, and the output variable is the categorical “low LNR/high LNR,” the random forest algorithm proved to be an appropriate choice. Random forest algorithms create a collection of random trees, and an optimized forest algorithm identifies the best subforest from this collection.^[35]^Furthermore, the random forest approach enables the assessment of variable importance and the analysis of the relationship between errors and the number of trees.

To address potential collinearity issues, LASSO regression is a particularly effective tool. This method incorporates variable selection by applying penalties to reduce collinearity. In the LASSO model, variables are penalized to varying degrees, those deemed more important receive lighter penalties and are more likely to be retained, whereas less significant variables face heavier penalties and are often excluded. This approach allows for the identification of the most critical prognostic factors, which can then be used to develop predictive models.^[36,37]^Moreover, machine learning methods can simultaneously process a large number of clinical, imaging, and pathological variables, which may be overlooked by human clinicians when handling such complex data.^[38]^ Human judgment can be influenced by experience, subjective preferences, or cognitive biases, whereas machine learning models make predictions based on objective patterns from the entire dataset.^[39]^ In addition, complex nonlinear relationships and interactions may exist between lymph node involvement and survival risk; traditional statistical methods or clinical experience may fail to fully capture these patterns, but machine learning can model them to improve predictive accuracy.^[40]^ Importantly, machine learning predictions are standardized and reproducible, whereas human assessments may vary across different clinicians or time points.^[41]^

This study’s retrospective design introduces several limitations. Firstly, inherent biases in observational research, such as confounding factors like EBV infection, may affect the outcomes of NPC modeling. Additionally, incomplete records of chemotherapy and surgical procedures within the SEER database represent another significant limitation that could impact the study’s results. Important details regarding chemotherapy, targeted therapy, medication regimens, and treatment durations were also not documented in the SEER database. The high rate of underreported chemotherapy cases further complicates result interpretation. A marked difference in mortality rates was observed between patients diagnosed before and after 2010, with a significant decline in mortality among the latter group. However, this improvement was not observed in patients with high LNR, possibly reflecting advances in surgical techniques and treatment protocols. Therefore, while our findings provide valuable insights, their generalizability may be limited due to the exclusive reliance on SEER data. The absence of validation using samples from different regions and countries is a notable limitation. Expanding the study to include data from diverse geographical locations would strengthen the robustness and accuracy of the findings.

## 5. Conclusion

In conclusion, our findings indicate an upward trend in the survival rates of NPC patients over time, highlighting progress in patient management strategies. However, despite these overall improvements, patients with high LNR continue to face a relatively poor survival prognosis. This highlights the need for exploring alternative treatment options to enhance survival outcomes in this particular subgroup. Continued research and targeted clinical interventions are essential to address the unique challenges posed by high LNR and to improve the overall prognosis for patients with NPC.

## Acknowledgments

We gratefully acknowledge the support and contributions of all individuals and organizations involved in this research.

## Author contributions

**Conceptualization:** Hongming Liao.

**Methodology:** Hongming Liao.

**Software:** Hongming Liao.

**Validation:** Hongming Liao, Benchao He.

**Writing – original draft:** Hongming Liao.

**Writing – review & editing:** Hongming Liao, Fengbo Yan.
